# The Technique of Mobilization of the Colon for Pull-Through Procedure in Hirschsprung’s Disease

**DOI:** 10.3389/fsurg.2022.921789

**Published:** 2022-07-05

**Authors:** Ruslan Bilal, Marat Ospanov, Damir Dzhenalayev, Yuri Olkhovik, Medet Khamitov, Arman Kozhakhmetov, Rauan Satbekov, Dina Abetova

**Affiliations:** ^1^ Department of Medicine, School of Medicine, Nazarbayev University, Nur-Sultan, Kazakhstan; ^2^ Department of Pediatric Surgery, National Research Mother and Child Center, Nur-Sultan, Kazakhstan; ^3^ Department of Pediatric Surgery, Medical University Astana, Nur-Sultan, Kazakhstan

**Keywords:** Hirschsprung’s disease, mobilization, blood vessels, pull-through, children

## Abstract

**Background:**

In patients with Hirschsprung's disease (HD), persistent obstructive symptoms may develop after surgery. The causes of mechanical obstruction may be a retraction of the pulled-through bowel due to insufficient mobilization of the mesentery or impaired blood supply in the area of the coloanal anastomosis in the case of excessive ligation of the supplying vessels. Leakage and stenosis are formidable complications and require repeated surgical intervention.

**Objective:**

The purpose of this study is to describe our experience and short-term results of the developed method: mobilization of the descending colon for its pull-through during the surgical treatment of the children with HD, which allows ensuring good mobility to the descending colon and maintaining blood supply at the same time.

**Methods:**

The medical records of 20 patients with rectosigmoid HD, who underwent parietal mobilization of the descending colon with preservation of the marginal artery, sigmoid arteries, and ligation of the left colon artery, were reviewed. This method is aimed at increasing the mobility of the brought-down bowel and maintaining the blood supply to the distal part of the brought-down bowel not only from the superior mesenteric artery but also from the lower one.

The SPSS Statistics 26.0 package was used for statistical analysis. To describe the analyzed data with a normal distribution, the mean values and the error of the mean were used. To determine the differences, Student’s *t*-test was used, and the differences were considered significant at a significance level of *p* < 0.05.

**Results:**

In all patients, the postoperative period was uneventful, without complications. The patients were discharged for outpatient treatment on average on the seventh day after the surgery. In all cases, there were no signs of anastomotic leakage or stricture on follow-up after 6–12 months.

**Conclusion:**

The method of mobilization of the colon in the rectosigmoid form of HD, parietal mobilization of the descending part of the colon preserving the marginal artery, sigmoid arteries, and ligating the left colon artery, can reduce the risk of complications by eliminating the tension of the descending colon.

## Introduction

Hirschsprung’s disease (HD) is a congenital anomaly characterized by the impaired motor function of the colon due to the presence of an aganglionic zone. The main method of treatment for this pathology is surgery, which consists of removing the affected segment of the colon and pulling down the ganglionic colon with the creation of a coloanal anastomosis (1). Despite the variety of methods for surgical treatment of HD (2–5), patients experience mechanical obstructive symptoms and coloanal anastomosis failure associated with the tension of the reduced bowel and impaired blood supply to the coloanal anastomosis zone (6–9). The frequency of strictures after surgery for HD reaches 17% of observations, the level of anastomotic leakage ranges from 3% to 7% of cases, and the formation of an abscess is observed up to 2%–6% (10–13). Complications require repeated surgical interventions in up to 11% of cases (13–18). In turn, reoperations are associated with a high degree of contamination and fecal incontinence (15, 18).

Usually, when mobilizing the descending colon to ensure its greater mobility, the colica sinistra (artery colica sinistra) or the inferior mesenteric artery is forcedly ligated (4, 5, 19). As a result, the inferior mesenteric artery ceases to participate in the blood supply of the colon. The blood supply to the distal part of the reduced intestine is provided only by the colica media, which causes the risk of necrosis or chronic hypoxia of the intestinal wall (6, 7, 19). If the colica sinistra (a. colica sinistra) is preserved, it is almost impossible to eliminate mesenteric tension. In turn, the tension of the mesentery or the colon itself can be a cause of retraction, anastomotic leakage or fixed bowel deformation, and stenosis of the coloanal anastomosis zone. Thus, the search for ways to bring down the descending part of the colon while maintaining an adequate blood supply is important to reduce the risk of complications. When we carried out a literature search in databases Google Scholar and PubMed, we did not find ways to mobilize the descending part of the colon that would increase its mobility and at the same time preserve collateral blood supply from a. inferior mesenteric in children with HD.

This publication presents a case series of 20 HD patients who underwent surgery to pull-through the descending colon segment using an alternative bowel mobilization technique.

## Methods

Medical records of 20 patients with rectosigmoid HD who underwent pull-through of the descending colon between 2017 and 2020 using an alternative bowel mobilization technique were analyzed retrospectively.

The SPSS Statistics 26.0 package was used for statistical analysis. To describe the analyzed data with a normal distribution, the mean values and the error of the mean were used. To determine the differences, Student's *t*-test was used, and the differences were considered significant at a significance level of *p* < 0.05.

### Ethical Approval

This retrospective study was approved by the local institutional council of Astana Medical University. All procedures performed in human studies were in accordance with the ethical standards of the local Institutional Ethics Committee of the Astana Medical University and the 1964 Declaration of Helsinki and its later amendments or comparable ethical standards.

Written consent was obtained from all (parents) of the individual participants.

### Description of the Procedure

**Figure 1:**
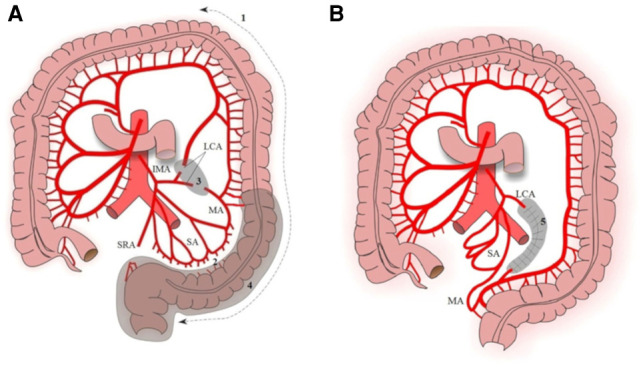
Mobilization of the colon. (**A**) Step 1, mobilization of the splenic flexure, descending colon, and sigmoid to the recto anal junction; step 2, mobilization of the marginal artery from the sigmoid colon; step 3, LCA division and formation of a “window” in the mesentery; step 4, colon pull-through. (**B**) Step 5, the formed mesentery “window” is sutured. SA, sigmoid arteries; LCA, left colic artery; IMA, inferior mesenteric artery; MA, marginal artery; SRA, superior rectal artery.

**Figure 2:**
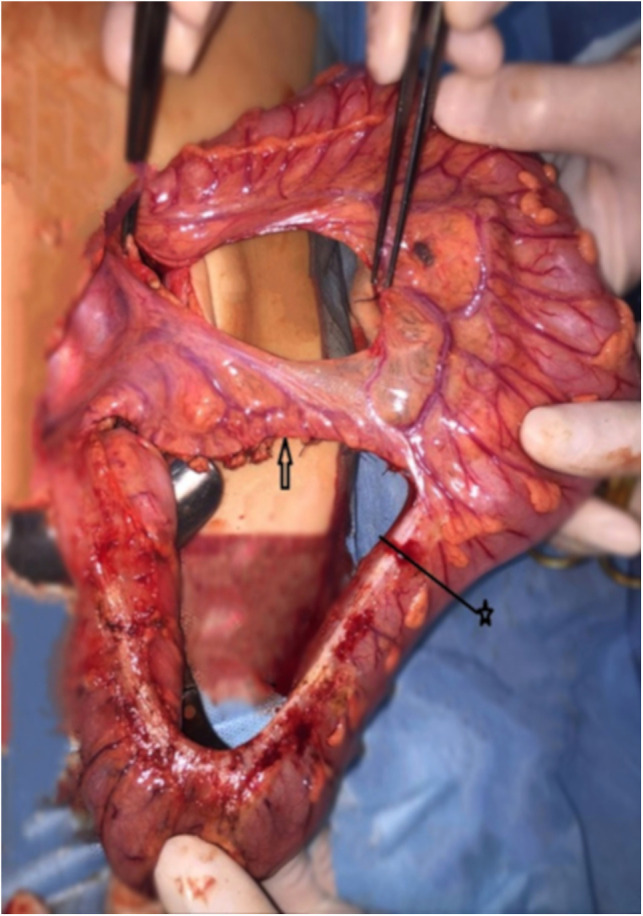
The arrow indicates the preserved sigmoid arteries. The tweezers indicate ligated left colic artery. The distance between the ends of the tweezers indicates how much bowel is free. ★—border of the colon resection.

Step 1: mobilization of the splenic flexure, descending colon, and sigmoid to the rectoanal junction (Figure. 1A).
Step 2: mobilization of the marginal artery from the sigmoid colon along the wall of the colon using Liga Sure, preserving the sigmoid arteries (Figure. 1A). The superior rectal artery also was divided.
Step 3: division of the left colic artery (LCA) and the formation of a “window” of the mesentery. To eliminate the tension, the left colic artery was dissected. After ligating and dividing the left colon artery, a “window” appears there in the mesentery. The “window” is expanded by dissection of the mesentery without reaching the adjacent arteries. This manipulation provides additional mobility (about 11.5 cm) to perform a pull-through of the descending colon (Figure. 2).
Step 4: colon pull-through.
Step 5: the formed mesentery “window” is sutured to prevent internal hernia (Figure. 1B).

An important factor is the preservation of sigmoid arteries, which provide collateral blood supply to the distal part of the reduced intestine from the inferior mesenteric artery.

We should pay attention to the fact that an increase in the length of the sigmoid arteries is achieved by ligation of the marginal artery from the colon. A preserved marginal artery connects the sigmoid arteries to the left branch of a. colica media.

The formed full-blooded, sufficiently long transplant with blood supply from the upper and the lower mesenteric artery is pulled-through freely, without the tension of coloanal anastomosis (Table 1). The feeding vessels are located quite close to the zone of coloanal anastomosis (Figures 1В and 3).

**Figure 3:**
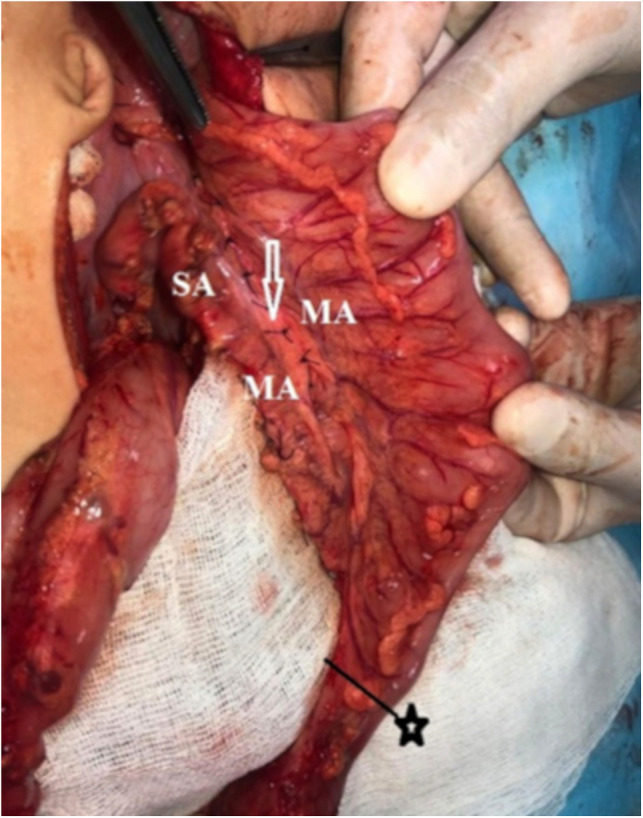
MA, marginal artery; SA, sigmoid arteries. The lengthening of the sigmoid arteries occurs due to the mobilization of the marginal artery. The arrow indicates the peritonization of the “window”. ★—border of colon resection.

**Table 1:** Increase in the length of the colon after each step of mobilization.

	Before crossing ACS (*n* = 20)	After crossing ACS (*n* = 20)	*р*
Average increase in bowel length after each dissection step, cm	4.3 ± 1.2	12.5 ± 2.3	0.01

*ACS, a. colica sinistra; n, number of patients.*

The procedure is completed with proctectomy and coloanal anastomosis creation using the Swenson technique with a single-layered interrupted suture.

### Bowel Length Measurement Technique

The inferior margin of the pubic symphysis is defined as the measurement reference point (coloanal anastomosis level). Before carrying out mobilization of the intestine, the distance from the site of the proposed resection of the colon to the lower edge of the pubic symphysis was measured. After each surgical step, the descending part of the colon was directed toward the symphysis of the pubis above the skin surface to determine the required length for the coloanal anastomosis. The distance from the site of the proposed resection of the colon to the lower edge of the pubic symphysis was recorded in centimeters using a ruler. The distance was recorded as negative if the proximal part of the colon did not exceed the lower edge of the symphysis and was marked as positive if the proximal part of the colon reached the lower edge of the symphysis (20). After crossing the lower branch or the main branch of a. colica sinistra the distance between the crossed ends of the vessels was additionally measured using a ruler.

### Clinical Outcomes

This operation was performed on 20 children. Eighteen of them were boys, and two were girls. The age of the children at the moment of the operation was between 6 months and 5 years. The average age was 2 years. The weight was from 7.5 to 20 kg. After the operation, enteral feeding was started on average on the third day, and the feces after the operation were observed on average on the second day. The early postoperative period in all 20 children was uneventful, without complications. The patients were discharged on the seventh day. The absence of obstruction was confirmed on follow-ups in 1, 6, 12, and 24 months. The follow-ups included rectal examination under anesthesia and a colon contrast study. Overall bowel function of the patients is as follows:—five children have mild encopresis, and one child has constipation due to megacolon. There was no retraction of the reduced bowel or stenosis of the coloanal anastomosis zone in any case.

## Discussion

Historical analysis of the implementation of the standard technique of bowel mobilization during operations according to the Duhamel, Svenson, Soave, and Rebein methods shows that the frequency of anastomotic leakage reaches 3%–7%, stenoses reaches 5%–24%, and abscesses reaches 2%–6% (21–25). Our study shows excellent short- and medium-term results after colonic descent using an alternative technique for mobilizing the vessels of the colon—parietal mobilization of the descending colon with preservation of the marginal artery, sigmoid arteries, and ligation of the left colon artery.

Nowadays, the Georgeson procedure is undoubtedly a gold standard for HD treatment. This procedure is performed in our hospital. Before doing the procedure laparoscopically, we had an objective to succeed in performing it with an open approach. We are planning now to perform it laparoscopically.

The developed method of mobilization of the colon can be a safe and effective alternative for the surgical treatment of patients with a high rectosigmoid transition zone with HD to prevent bowel tension and impaired blood supply to the intestine.

Usually, in children with HD with a high rectosigmoid transition zone, the superior rectal and sigmoid arteries are transected. To mobilize the descending colon, the left colic artery (a. colica sinistra) or the inferior mesenteric artery (inferior mesenteric artery) is forcedly ligated (4, 5, 19). As a result, the inferior mesenteric artery ceases to participate in the blood supply to the colon. The blood supply to the distal part of the reduced bowel is provided only by the middle colon artery (a. colica media), which causes the risk of developing necrosis, anastomotic leakage, or chronic hypoxia of the colonic wall (6–8, 19). Chronic hypoxia, in turn, is the cause of acquired aganglionosis (8, 19, 26) or hypogangliosis (27).

Insufficient mobilization of the mesentery of the colon can lead to tension with impaired blood supply to the corresponding colon segment of 5.8% (7). The tension of the mesentery or the bowel itself can cause anastomotic leakage of 3%, bowel necrosis of 2.4%, fixed bowel deformity of 1.2%, or stenosis of the coloanal anastomosis zone of 23% (7).

There were no obstructive symptoms in this series of patients after surgery.

## Data Availability

The original contributions presented in the study are included in the article/Supplementary Material, further inquiries can be directed to the corresponding author/s.
